# Efficacy of the Essential Amino Acids and Keto-Analogues on the CKD progression rate in real practice in Russia - city nephrology registry data for outpatient clinic

**DOI:** 10.1186/s12882-016-0281-z

**Published:** 2016-07-07

**Authors:** Alexander Zemchenkov, Irina N. Konakova

**Affiliations:** City Mariinsky Hospital – City Nephrology Center, Liteiny pr., 56, 191104 Saint Petersburg, Russian Federation; North-Western State Medical University n.a. I.I. Mechnikov, Internal Diseases and Nephrology Chair, Kirochnaya str., 41, 191015 Saint Petersburg, Russian Federation; First St.-Petersburg State Medical University n.a. I.P.Pavlov, Nephrology and Dialysis Chair, L’va Tolstogo str., 6-8, 197022 Saint Petersburg, Russian Federation

**Keywords:** CKD progression, Low protein diet, Supplemented low protein diet, Essential Amino Acids and Keto-Analogues, eGFR slope

## Abstract

**Background:**

Renal replacement therapy (RRT) is growing by 10 % per year in Russia, but pre-dialysis care which can retard CKD progression and delay the start of RRT remains limited. We evaluate the effect of Essential Amino Acids and Keto-analogues (EAA/KA) on CKD progression.

**Methods:**

The effect of low protein diet (LPD), supplemented by EAA/KA, on GFR slope changes between first and second treatment period (five sequential visits per period) in 96 patients withs CKD Stage 3B-5 was compared to GFR slope changes in the control group of 96 patients, randomly selected from matched (by gender, age, diagnosis and CKD Stage) cohort of 320 patients from the city Registry. The mean baseline eGFR was 23 ± 9 ml/min/1.73 m2; 29 % had CKD3B, 45 % - CKD4, 26 % - CKD5.

**Results:**

The rate of eGFR decline changed from −2.71 ± 2.38 to −2.01 ± 2.26 ml/min/1.73 m2 per year in the treatment group and from −2.18 ± 2.01 to −2.04 ± 2.18 ml/min/1.73 m2 per year in the control group. Only in the treatment group the difference was significant (*p* = 0.04 and *p* = 0.6). Standardized effect size for intervention was significant in treatment group: −0.3 (of pooled SD), 95 % CI −0.58 ÷  −0.02 and non-significant in control group: −0.07 (−0.35 ÷ +0.22). The univariate and multivariate analysis of EAA/KA therapy effect demonstrated that it was probably more effective in patients of older age, with higher time-averaged proteinuria (PU), lower phosphate level, in patients with glomerular v. interstitial diseases, and in females. Only the latter factor was significant at pre-specified level (<0.05).

**Conclusions:**

LPD combined with EAA/KA supplementation lead to the decrease of the CKD progression both in well-designed clinical study and in real nephrology practice in wide variety diseases and settings. Registry data can be helpful to reveal patients with optimal chances for beneficial effect of LPD supplemented by EAA/KA.

**Trial registration:**

ISRCTN28190556 06/05/2016.

## Background

Renal replacement therapy (RRT) is growing rapidly in Russia with average 10 % increase per year [1]. That reflects the increasing number of new dialysis centers (mainly private, erected by international dialysis networks) in substantially changing conditions, including implementation of diagnosis-related groups (DRG) system for reimbursement [2]. Prevalence of RRT remains rather low, however it grew from 56 per million population (pmp) in 1998 to 246 pmp in 2013 (Russian Dialysis Society Registry data [1]) and 299 pmp in 2015 (marketing data). It means that dialysis treatment becoming now available for majority of patients in most regions of Russia, the more so as the average incidence rate remains rather low – 51 pmp, presumably due to younger population, lower prevalence of diabetes mellitus, lower and later CKD revealing. Nevertheless, some regions, like Saint Petersburg [3] (70 pmp) look similar to the neighboring countries like Finland (89 pmp) and Estonia (63 pmp). The proportion of patients with functioning graft among those on RRT decreased slowly from 25 % in 1998 to 19 % in 2013, while the proportion of HD patients increased from 70 % in 1998 to 75 % in 2013. The proportion of PD patients remains stable low - 5–7 %. Thus low-protein diet (LPD) actually is no more considered as the method to ensure survival while waiting period for RRT, but the issue of quality of life, retardation of CKD progression and delaying “the health start” of dialysis.

Requirements for dietary protein intake in the non-nephrotic CKD patients without inflammatory conditions or other catabolic states appear to be equal to the requirements of healthy individuals: average 0.6 g/kg/day of unselected or mixed biological value [4].

Regimens with less than 0.6 g/kg/day protein intake are often difficult for application, unless ‘non-proteic’ (commercially available) carbohydrates are applied [5] to maintain enough calorie consumption, however this products are not easily available everywhere for every patient and different approaches are acceptable [6].

The rate of CKD progression, evaluated by decrease of estimated GFR (CKD-EPI equation) depends on numerous factors and can be delayed by nephroprotective therapy, in particular – by nutrition therapy. We assessed the effect of LPD supplemented by keto-analogues (EAA/KA) therapy on the rate of eGFR decline in the real practice, using prospectively collected data obtained from St.-Petersburg city nephrology Registry.

## Methods

All patients with CKD Stage 3–5, referred to city nephrology center, are offered dietary counseling by experienced nephrologist. LPD is routinely recommended to all patients at high risk for CKD progression after evaluation the CKD stage, GFR decline rate and excluding the symptoms and signs of protein-energy wasting based on physician’s judgment and labs: albumin <3.8 or <3.5 g/dl for diabetics, phosphate < 0.8 mmol/l; in some cases anthropometric data (skinfold, calculated muscle section area) and additional criteria (absolute lymphocyte number, transferrin level) are used. Patients with very low life expectancy are excluded from low protein diet interventions. Detailed nutritional manual for patients with CKD is available for patients [7]. Several examples of daily LPD (0.6 g/kg/day) and VLPD (0.3 g/kg/day) adopted for Russian traditions and food habits are presented in the Appendix. Patients can get information about exchangeability of some products, about forbidden and allowed products.

Patients were initially prescribed a standard LPD (0.60 g protein/kg body weight/day). For patients who demonstrated treatment compliance to LPD, low-phosphate diet, nephroprotective therapy (iACE or ARB), and have moderate to severe proteinuria (PU) – defined as PU >1.0 g/day, the additional restriction of dietary protein, supplemented by EAA/KA may be considered. Patients are provided with EAA/KA (prescribed dose - one pill per 5 kg body weight) by the special social drugstore, supplied from budgetary funded source, so-called “additional medicinal providing”. About 200 patients simultaneously receive EAA/KA in St.-Petersburg from this source. For logistics, clinical and personal reasons not all patients have received EAA/KA without substantial interruptions and long enough to evaluate the changes in GFR slope.

Patients, comprising study group were selected from St-Petersburg CKD registry (which includes more than 6000 patients with CKD3+ as we described in details earlier [3]) according following ***criteria:*** confirmed moderate GFR decline rate (see below); patient’s compliance to diet and pharmacological therapy; and prolonged history of regular EAA/KA therapy according to the data from special database, recording patient’s’ visits to drugstore for EAA/KA supplied by budgetary funded source (≥10 consecutive visits with pre-defined for each CKD stage frequency). Patients, who received commercially available EAA/KA were not included in the study as we could not ensure adequate treatment regimens. In 96 patients with CKD stages 3B-5 the progressive decrease of eGFR was confirmed by previous monitoring during > 6 months. GFR decrease rate was evaluated as linear regression coefficient of CKD-EPI GFR versus visit date with the number of visit ≥ 4 (median 5; IR 4 ÷ 7). Patients with rapid CKD progression (more than 10 ml/min/1.73 m^2^ per year) were excluded from analysis. The mean baseline eGFR was 23 ± 9 ml/min/1.73 m^2^; 29 % of patients had CKD3B, 45 % - CKD4, 26 % - CKD5. We intend to maintain all patients on LPD with protein intake 0.8–0.6 g/day. Patients receiving EAA/KA therapy are advised to keep to LPD with protein intake < 0.6 g/day (evaluated by 3-day diaries). Protein-free products are included to supply energy with negligible load of phosphorus, sodium, potassium and nitrogen [8].

Evaluation of treatment compliance for the diet is more qualitative than quantitative. We request our patients to provide food diaries at least for three days between visits at every visit. The most concerned patients provide diaries for all period between visits. During the visit nephrologist can discuss the patient’s menu while labs are processed, as unfortunately patients have no regular access to dietician consult. LPD have to provide at least 30 kcal/kg/day of energy, lower intake of calories is considered in those with body mass index (BMI) >28 kg/m^2^. Phosphate binders (calcium carbonate or calcium acetate or aluminum hydroxide) are prescribed according to the guidelines in order to maintain serum phosphate within the normal range (0.81–1.45 mmol/l) [9]. As a nephroprotective and antihypertensive therapy 64 % of patients received ACEi, 16 % - ARB, 24 % - CCB, 11 % - beta-blockers.

Patients keeping diet undergo full clinical evaluation, including dietary counseling with special emphasis to diet adherence at least quarterly for CKD3, every 2 months for CKD4, and monthly for CKD5. At each visit, blood pressure (BP), body weight, serum urea, creatinine, sodium, potassium, phosphate, calcium, total protein, albumin, and hemoglobin are measured; transferrin saturation and ferritin are measured quarterly, total cholesterol, triglycerides – twice a year. In patients with more often visits, additional visit data was averaged with those of the closest visit.

### Statistical analysis

Continuous variables were presented as the mean ± standard deviation (SD) – for normally distributed variables - or as median (25 ÷ 75 percentiles) for others. Categorical data were presented as percent of frequency. The comparisons between normally distributed variables were performed by Student test, between other continuous variables – by Mann–Whitney test, between categorical parameters – by *χ*^2^ test with *P*-values <0,05 for significance. The relationship between continuous variables was investigated by the Pearson correlation coefficients and *P*-values. The size effect was evaluated by raw paired difference and by paired difference standardized by standard deviation and corresponding 95 % confidence interval.

## Results

### Evaluation of effect in treatment group

The effects of LPD supplemented by EAA/KA were evaluated during period of 10 routine visits to nephrologist following the EAA/KA prescription. The mean dose of EAA/KA was 11 ± 2 pills per day (0.8 ± 0.1 pills/5 kg BW – 20 % lower than prescribed). The slope of the eGFR decrease was calculated by regression model for first 5 visits and for following 5 visits. These data were compared to the data for 96 patients, randomly selected from matched group (by gender, age, diagnosis, proteinuria and CKD stage) of 320 patients from the registry. Table 1 shows comparison of baseline parameters in two groups.

**Table 1:** Baseline characteristic of patients in treatment and control group

	Treatment group	Control group	*p*
Number of patients	96	96	
Diabetes mellitus type II	12/96 (13 %)	14/96 (14 %)	*p* = 0.8
Polycystic kidney disease	6/96 (6 %)	8/96 (8 %)	*p* = 0.6
Hypertension	23 (24 %)	20 (21 %)	*p* = 0.6
Male/female	40/56 (42 %)	44/52 (46 %)	*p* = 0.6
Age	54 ± 15	57 ± 13	*p* = 0.14
eGFR (CKD-EPI), ml/min/1.73 m^2^	23 ± 9	25 ± 11	*p* = 0.17
CKD stage 3B/4/5	28/43/25 (29 %/45 %/26 %)	33/37/26 (34 %/39 %/27 %)	*p* = 0.6
Albumin, g/dl	4.1 ± 0.5	4.2 ± 0.4	*p* = 0.13
Calcium, mmol/l	2.28 ± 0.16	2.26 ± 0.14	*p* = 0.4
Phosphate, mmol/l	1,27 ± 0,26	1,21 ± 0,24	*p* = 0.10
Sodium, mmol/l	140 ± 3	141 ± 3	*p* = 0.02
Cholesterol, mmol/l	5.8 ± 1.2	5.5 ± 1.0	*p* = 0.06
Triglycerides, mmol/	2.06 ± 0.98	1.85 ± 1.11	*p* = 0.19
Hemoglobin, g/dl	12.4 ± 1.4	12.2 ± 1.3	*p* = 0.3
Serum Fe, μmol/l	13 ± 5	12 ± 4	*p* = 0.13
CRP, mg/dl	0.1 ÷ 0.6	0.2 ÷ 0.8	*p* = 0.13
Mean proteinuria, g/day	1.18 ÷ 2.56	1.24 ÷ 2.42	*p* = 0.06
Maximal proteinuria, g/day	1.49 ÷ 4.94	1.56 ÷ 5.72	*p* = 0.11
Number of patients with hypertension (>140/90 mmHg)	31/96 (32 %)	34/96 (35 %)	*p* = 0.5
Systolic blood pressure	137 ± 20	139 ± 22	*p* = 0.5
Diastolic blood pressure	83 ± 12	81 ± 11	*p* = 0.2

The only statistically significant (but not clinically significant) was the difference in serum sodium level. Table 2 shows that the comorbidity in treatment and control groups was similar.

**Table 2:** Charlson comorbidity index in treatment and control groups (M ± SD or number of cases)

	Treatment group	Control group	*p*
Charlson comorbidity index	4,91 ± 1,67	5,09 ± 1,58	0,59
Myocardial infarction	8	9	0,73
Congestive heart failure	24	28	0,37
Peripheral disease	43	36	0,14
Cerebrovascular disease	28	24	0,35
Dementia	3	6	0,21
Chronic pulmonary disease	9	13	0,23
Connective tissue disease	2	5	0,17
Peptic ulcer disease	13	19	0,12
Mild liver disease	11	18	0,07
Diabetes without end-organ damage	10	11	0,75
Hemiplegia	4	2	0,15
Moderate or severe renal disease	96	96	–
Diabetes with end-organ damage	2	3	0,56
Tumor without metastasis	4	3	0,61
Leukemia	1	1	–
Lymphoma	1	1	–
Moderate or severe liver disease	3	4	0,61
Metastatic solid tumor	0	0	–
AIDS	0	0	–

Table 3 shows the changes of eGFR during first and second periods of the study in treatment group vs control group. The duration of the first and second five-visit period was 8.6 ± 1.6 and 8.4 ± 1.9 months for treatment group and 8.9 ± 2.0 and 8.6 ± 2.0 months for control group (all differences are non-significant, *p* > 0.05).

**Table 3:** The size effect of intervention: difference in eGFR decline rate between first and second period of study in treatment group vs control group

	eGFR decline rate		Standardized effect size		
	First 5 visits	Second 5 visits	Bias corrected (Hedges)	Standard error of effect size estimate	Confidence interval for effect size
	ml/min/1.73 m^2^ per year				
Treatment group	−2.71 ± 2.38	−2.01 ± 2.26	0.30	0.15	0.02÷0.58
Control group	−2.18 ± 2.01	−2.04 ± 2.18	0.07	0.14	−0.22÷0.35
Difference	*p* = 0.10	*p* = 0.93	*p* < 0.001		

The rate of eGFR decline changed from −2.71 ± 2.38 to −2.01 ± 2.26 ml/min/1.73 m^2^ per year in the treatment group and from −2.18 ± 2.01 to −2.04 ± 2.18 ml/min/1.73 m^2^ per year in the control group. While decrease of the eGFR decline was noticed in both treatment (−0.70 (0.04 ÷ 1.36; ml/min per year) and control (−0.14 (−0.46 ÷ 0.74;) groups, only in the treatment group this difference was significant (*p* = 0.04 and *p* = 0.6). The difference in raw effect size was −0.56 ± 0.10 ml/min/1.73 m^2^ per year, (95 % confidential interval −0.75 ÷ −0.37), standardized effect size for intervention was significant in treatment group: −0.3 (of pooled SD), 95 % CI −0.58 ÷  −0.02 and non-significant in control group: −0.07 (−0.35 ÷ +0.22).

### The search for pro- and con- factors for EAA/KA effect

Table 4 demonstrates the results of univariate and multivariate analysis of EAA/KA therapy effect in association with some potential confounding parameters. For categorical (binary) variables the comparison of the changes (from first to second period) of GFR slope are presented for two levels of binary variable. Table 3 also shows the results of multi-variate analysis of EAA/KA therapy. This intervention was probably more effective in patients of older age, with greater time-averaged PU, lower phosphate level, in glomerular v. intestinal diseases, and in females (p for exclusion from regression model 0.10). Only the latter factor was significant at pre-specified level (<0.05).

**Table 4:** Reducing effect of EAA/KA on GFR slope decrease: the results of uni-variate analysis, categorical variable comparison and variables in final multi-variate model

	Uni-variate analysis			Final model of multi-variate analysis^b^		
Variable	Unstandardized B	*p*	95 % CI for B	Unstandardized B	*p*	95 % CI for B
Age (per 10 year)	0.023	0.08	−0.004 ÷ 0.05	0.021	0.06	−0.002 ÷ 0.04
PU mean (per 1 g/day)	−0.061	0.08	−0.13 ÷ 0.09	−0.065	0.06	−0.14 ÷ 0.005
PU max (per 1 g/day)	−0.023	0.38	−0.004 ÷ 0.05	–	–	–
Albumin (per 0.1 g/dl)	−0.061	0.21	−0.04 ÷ 0.16	–	–	–
Phosphate (per 0.1 mmol/l)	0.015	0.18	−0.008 ÷ 0.04	0.016	0.08	−0.003 ÷ 0.03
Hb (per 1 g/dl)	0.018	0.32	−0.02 ÷ 0.06	–	–	–
ln CRP (per 0.1 mg/dl^a^)	−0.039	0.14	−0.09 ÷ 0.15	–	–	–
Categorical variable comparisons				Categorical variable in equation		
Glom. v. interst.	−0.082 ± 0.035	0.06	−0.059 ± 0.044	−0.016	0.06	−0.003 ÷ 0.03
Female v. male	−0.079 ± 0.025	0.11	−0.066 ± 0.024	−0.013	0.03	−0.003 ÷ − 0.001
DM v. non-DM	−0.072 ± 0.021	0.50	−0.069 ± 0.023	–	–	–

*CI* confidence interval, *PU* proteinuria, g/day, *mean* averaged for study period, *max* maximum per study period, *Hb* hemoglobin, *CRP* C-reactive protein, glom. glomerular diseases, interst. interstitial diseases, *DM* diabetes mellitus

^a^Before ln-transformation

^b^p for exclusion from model 0.10

Although beneficial effect of therapy was not significantly linked to PU level in uni- and multivariate analysis, the slope of eGFR decline was not surprisingly directly associated with PU.

As PTH target ranges were determined at different levels in CKD3 (<70 pg/ml), CKD4 (<85 pg/ml) and CKD5 (non-dialysis - <110 pg/ml) in Russian National CKD-MBD guidelines, we evaluated the percentage of patients with PTH above these levels: 23/96 in treatment group and 26/96 in control group (*p* = 0.6); the mean baseline PTH levels were 69 ± 32 v. 63 ± 37 pg/ml (*p* = 0.4). The slope of PTH elevation was not significant in both groups and did not differ between the groups (2 ± 11 pg/ml/year, *p* = 0.1).

The difference in raw effect size was slightly more expressed in women (−0.58 ± 0.11 v. –0.54 ± 0.09 ml/min/1.73 m^2^ per year; *p* = 0.06) and in patients with glomerular diseases compared to interstitial diseases (−0.60 ± 0.15 v. –0.54 ± 0.14 ml/min/1.73 m^2^ per year; *p* < 0.01).

During the study period 26 patients from treatment group entered the more advanced CKD stage, in 62 patients CKD stage did not change, and 8 patients showed slow but stable improvement of kidney function; corresponding subgroups in control group consisted of 32, 57 and 7 patients respectively (for difference in *χ*^2^-test *p* > 0.2).

## Discussion

Comparing the changes of GFR slope from first to second period in the intervention (EAA/KA therapy) group and control group we demonstrated that LPD, supplemented by EAA/KA, could decrease the rate of CKD progression. Five-visit interval of evaluation for eGFR slope was rather long and different factors could interfere the CKD progression, however five visits data is necessary to calculate eGFR precisely enough to be reliably compared in various settings. Possibly the first period (as comparator) should be shorter to exclude the already achieved effect of EAA/KA therapy that mitigate the difference.

Chang et al. demonstrated similar results, in their study EAA/KA was added for 6 months to previously prescribed LPD (0.6 g/kg/day of proteins for 6 months) in 120 patients with CKD3-4. The decline of GFR slopes during the LPD + EAA/KA period was significantly lower than during the LPD alone period. Multivariate analysis revealed that responsiveness to LPD + EAA/KA was independently related to diabetes (*p* = 0.006) and high serum albumin levels (*p* = 0.011) in the LPD alone period [10]. The potentially influencing factors in our study (Table 3) were different, that may reflect the differences in the patient’s population. Di Iorio et al. demonstrated the influence of phosphate level in the study of 99 proteinuric CKD patients, transferred from LPD to very low protein diet (VLPD), which resulted in the halving of proteinuria, but the anti-proteinuric effect was attenuated by high level of phosphate [11].

In earlier (and smaller) prospective randomized study Teplan et al. demonstrated that LPD (0.6 g/kg/day of proteins) supplemented with EAA/KA led to lower decrease of kidney function in patients with baseline GFR 22–36 ml/min, compared to LPD alone, although the number of follow-up visits was very small (once in 6 months) [12].

Despite VLPDs are usually considered more effective in postponing dialysis in compliant patients, only minority (as low as 14 %) of patients can follow it properly [13], which was demonstrated by the data from the assessment period of randomized study with 3-year period of enrollment. After such strict selection VLPD group showed 57 % lower GFR decline rate (−4.9 v. -2.1 ml/min per year). Interestingly, we found very similar results in our supplemented LPD group (−2.01 ± 2.26). In above mentioned publication standard deviation for GFR decline rate was not presented, so it is not possible to compare standardized size effect of interventions.

Recent meta-analysis (7 randomized controlled trials, one cross-over trial, and one non-randomized concurrent control trial, all of them published before April 2015) results indicated that comparing to normal protein diet, LPD or VLPD supplemented with keto-analogues (SLPD/SVLPD) were able to significantly prevent the deterioration of kidney function, defined by eGFR (*P* < 0.001); hyperparathyroidism (*P* = 0.04); hypertension (*P* < 0.01); and hyperphosphatemia (*P* < 0.001) – thus could delay the progression of CKD effectively without causing malnutrition [14].

Supplementation with EAA/KA is not strictly needed for vegan diets with protein intake 0.6 g/kg/day, but if applied, EAA/KA ensure the balanced intake of different aminoacids. If EAA/KA are not used, different types of products (e.g. legumes and cereals) should be combined at every meal [15], that can be burdensome for some patients. Supplementation permits also a simplified approach, which just exclude animal products, but allows a free choice of vegetables, fruits, legumes and cereals [5, 6].

Additional approach to enhance the compliance to LPD - an occasional unrestricted meal (one to three times per week) - is practiced by at least two Italian groups of researchers [5], in elderly patients in particular [16]: Usually we do not practice broadly this approach as we consider that some aspects of Russian mentality will prompt our patients to subsequent diet liberalization. Nevertheless, we believe this approach could be useful for some patients.

As special protein-free food is virtually available in Russia (with one historical precursor – protein-free bread in 1980s), but the choice is limited (as some of them are very salty, and others are rather expensive or contain substantial amount of phosphate). More or less acceptable is mainly pasta of stretch origin, or mixtures for homemade bakery. Protein-free food is partly available for free to CKD patients only in Italy [17].

The MDRD Study and a secondary analysis of this study did not suggest a clear benefit from SVLPD as compared with the 0.60 g protein/kg/d, although there was a trend towards slower progression of kidney failure with the SVLPD. However, SVLPD used in the MDRD Study probably was not ideal because the keto acid/EAA supplements contained rather large amount of tryptophan, which could generate more nephrotoxic metabolites, particularly indoxyl sulfate [4]. Hence, it is possible that alternative keto acid/EAA supplements will be more effective in slowing of the CKD progression.

Presently only two of mixtures/combinations of amino acids and keto acids, both of which are marketed by the same company, are available: Alfa Kappa (in Italy) or Ketosteril (globally) [6].

Potential drawbacks come down when the vegetarian diet is associated with too restricted protein intake and/or insufficient energy intake, justifying an early and regular follow-up by a nephrologist to avoid malnutrition [15]. Unfortunately, due to lack of reimbursement for this service, we presently have no access to concealing our patients by certified dietitian on regular basis.

Although we have no rigorous confirmation for benefit of supplementation of LPD by EAA/KA in RCT, we have to keep in mind that as soon as the efficacy of intervention strongly depends on the patient’s active participation, RCT may be inappropriate tool because the random allocation per se can reduce the effect of the intervention [17]. The alternative approach is the search for the factors and conditions, which could make the intervention more efficient. Controlled observation studies are one of important step on this way. Various LPDs cannot only influence the CKD progression, but also impact on dialysis outcomes, when patients finally achieve CKD5D stage. In this area, the RCT’s are more unfeasible.

Bellizzi et all showed, that mortality risk in dialysis period was 0.59 (*p* < 0.001) after SVLPD pre-dialysis treatment, compared to propensity score matched group of unselected control, but risk for patients, previously treated with LPD was not different from SVLPD group (*RR* = 0.84; *p* = 0.496).In subgroup analysis females aged <70, without diabetes and cardiovascular diseases, had more pronounced treatment effect [18].

In recent study from Taiwan, Markov model showed that the group with EAA/KA early initiation gained higher QALYs with lower cost when compared to the watchful-waiting group. Analysis of sensitivity indicated that early EAA/KA initiation (eGFR 17–29 mL/min/1.73 m^2^) would be the preferred cost-effective option, if achieved relative reduction of eGFR decline, associated with LPD plus EAA/KA, is > 4 % [19]. Due to insufficient power our study we could not differentiate the size effect between different CKD stages, but could show that the LPD with EAA/KA supplementation resulted in 7 % relative reduction of eGFR decline rate (compared with control group) – thus, could be cost-effective.

Moderately restricted LPDs may be adapted to virtually any cuisine and should be tailored to the patients’ preferences, while VLPDs usually require trained, compliant patients; a broader offer of diet options may lead to more widespread use of LPDs [17].

### Limitations

The patient selection for LPD (<0.6 g/kg/day) supplemented by EAA/KA in our study was not random and could contain selection bias (patients with better compliance to diet can have better compliance to other nephroprotective strategies, such as antihypertensive and anti-RAAS therapy, sodium restriction, and optimal glycemic control. On the other hand, the matched control group recruited from 320 similar patients was rather close to the treatment group. These confounding factors were taken in consideration in multiple regression analysis. Of note, patients with pre-dialysis CKD were not regularly evaluated for acid-base balance, and rarely received bicarbonates; this information was not included into analysis. Evaluation of compliance to diet was more qualitative than quantitative, but such approach may be considered as acceptable [6]. The number of patients, included into analysis, was relatively small as it was restricted to those who received EAA/KA supplied from budgetary funded source; on the other hand, the amount of EAA/KA gained in drugstore was available as a surrogate measure for received doses in parallel with patients’ diary.

## Conclusions

Low protein diet combined with EAA/KA supplementation lead to the decrease of the CKD progression both in well-designed clinical study and in real nephrologist practice in wide variety diseases and settings. Registry data can reveal patients with optimal chance for beneficial effect of LPD supplemented by EAA/KA.

## Abbreviations

CKD, chronic kidney disease; CRP, C-reactive protein; DM, diabetes mellitus; DRG, diagnosis-related groups reimbursement system; pmp: per million population; EAA/KA, Essential Amino Acids and Keto-Analogues; eGFR, estimated glomerular filtration rate; HT, hypertension; LPD, low protein diet; PU, proteinuria; RRT, renal replacement therapy; SD, standard deviation; VLPD, very low protein diet

## Appendix

### The examples of daily LPD (0.6 g/kg/day) and VLPD (0.3 g/kg/day)

#### LPD (0.6 g/kg)

Day 1

- ***Breakfast 1.*** Bread, butter, honey, coffee.
  - (mixed bread 40 g, white bread 40 g, butter 20, honey 30 g, cream 10 ml, sugar 10 g).
- ***Breakfast 2.*** Applesauce.
  - (apple 100 g, sugar 10 g, cinnamon steak)
- ***Dinner.*** Quenelle, cauliflower, potato; chocolate jelly.
  - (pork 50 g, onion 20 g, bread 30 g, cauliflower 150 g, vegetable oil 10 g, corn starch 5 g, nutmeg, cream 10 ml, potato 200 g; cream 30 ml, water 100 ml, sugar 10 g, cocoa 5 g, gelatin 2 g).
- ***Lunch.*** Bread, butter, marmalade, coffee or tea.
  - (mixed bread 20 g, butter 10 g, marmalade 20 g, sugar 10 g).
- ***Supper***. Bread, butter, black pudding, celery salad, tea.
  - (mixed bread 80 g, butter 20 g, black pudding 30 g; celery 150 g, apple 50 g, vegetable oil 10 g, vinegar, onion, ginger, sugar 10 g).
- ***Energy****2250 kcal/9410 kJ; protein – 41 g, fat – 108 g, carbohydrates – 267 g, sodium – 0.74 g, potassium – 2.7 g, calcium – 0.27 g, phosphorus – 0.8 g.*

Day 2

- ***Breakfast 1.*** Bread, butter, marmalade, smoked sausage, coffee.
  - (mixed bread 40 g, white bread 40 g, marmalade 20 g, smoked sausage 30 g, butter 20, cream 10 ml, sugar 10 g).
- ***Breakfast 2.*** Bread, lard, onion, tea.
  - (mixed bread 40 g, lard 15 g, onion 5 g,,sugar 10 g).
- ***Dinner.*** Potato mash, boiled egg, sweet and sour sauce, salad.
  - (potato 250 g, cream 30 ml, butter 10 g, vegetable oil 10 g, 1 egg, lard 10 g, corn starch 10 g, onion 5 g, bay leaf, pepper; red beet – 100 g, vinegar, caraway-seeds onion 5 g, vegetable oil 5 g, sugar 10 g).
- ***Lunch.*** Bread, butter, marmalade, coffee.
  - (white bread 40 g, butter 10 g, marmalade 20 g, sugar 10 g).
- ***Supper***. Fried potato, vegetable aspic, tea.
  - (potato 200 g, butter 10 g, lard 10 g, onion 5 g, boiled carrot 100 g, green peas 30 g, cauliflower 30 g, water 120 g, onion 30 g, bay leaf, clove, mustard 5 g, vinegar).
- ***Energy****2260 kcal/9460 kJ; protein – 40 g, fat – 107 g, carbohydrates – 274 g, sodium – 1 g, potassium – 3.37 g, calcium – 0.24 g, phosphorus – 0.75 g.*

Day 3

- ***Breakfast 1.*** Bread, butter, marmalade, coffee.
  - (mixed bread 40 g, white bread 40 g, marmalade 30 g,, butter 20, cream 10 ml, sugar 10 g).
- ***Breakfast 2.*** Fruit salad.
  - (apple 50 g, cherry 50 g, lemon juice 10 g, sugar 15 g).
- ***Dinner.*** Chicken with sauce, rice, salad, currant pudding with cream.
  - (chicken breast 60 g, mushroom 20 ml, onion 20 g, apple 50 g, vegetable oil 10 g, rice 50 g, butter 10 g, pet Sai 80 g, cream 10 g, vinegar; red currant juice– 100 ml, water 50 ml, sugar 10 g, corn starch 10 g, cream 10 g).
- ***Lunch.*** stick-like biscuits 20 g, butter, marmalade, coffee.
  - (white bread 40 g, butter 10 g, honey 20 g, sugar 10 g, cream 10 g.).
- ***Supper***. Scrambled eggs with tomato, herb butter, tea.
  - (1 small egg, tomato 30 g, butter 10 g, lard 10 g, onion 5 g, mixed bread 80 g, butter 20, sugar 10 g).
- ***Energy****2279 kcal/9450 kJ; protein – 40 g, fat – 106 g, carbohydrates – 275 g, sodium – 1.3 g, potassium – 055 g, calcium – 0.19 g, phosphorus – 0.67 g.*

#### VLPD (0.3 g/kg/day)

Day 1

- ***Breakfast 1.*** Bread, butter, honey, marmalade, coffee.
  - (low-protein bread 80 g, butter 10, marmalade 20 g, honey 10 g, cream 10 ml, sugar 10 g).
- ***Breakfast 2.*** Sago.
  - (fruit juice 150 ml, water 50 ml, sago 20 g, sugar 15 g).
- ***Dinner.*** Potato, poached egg, apple compote.
  - (potato 300 g, 1 small egg, butter 20 g, tomato 20 g,; apple 100 g, sugar 10 g, cream 20 g).
- ***Lunch.*** Toast, butter, cream, coffee or tea.
  - (low-protein toast 40 g, butter 15 g, cream 10 g, sugar 5 g).
- ***Supper***. Rice salad, bread, butter, tea.
  - (rice 20 g, onion 10 g, carrot 30 g, mushroom 40 g, mayonnaise 30 g, lemon juice 5 g; low-protein bread 60 g, butter 20 g, sugar 15 g.)
- ***Energy****2336 kcal/9782 kJ; protein – 24 g, fat – 115 g, carbohydrates – 293 g, sodium – 1.65 g, potassium – 2.36 g, calcium – 0.19 g, phosphorus – 0.48 g.*

Day 2

- ***Breakfast 1.*** Bread, butter, honey, marmalade, coffee.
  - (low-protein bread 80 g, butter 20, marmalade 20 g, honey 15 g, cream 10 ml, sugar 10 g).
- ***Breakfast 2.*** Bread, butter, cheese, cucumber, tea.
  - (low-protein bread 40 g,, butter 10 g, cheese 10 g, cucumber 30 g, sugar 5 g).
- ***Dinner.*** Potato dumplings, plum compote.
  - (potato 250 g, 1 small egg, potato stretch 40 g, butter 30 g; plum compote 150 g).
- ***Lunch.*** Cream cocktail.
  - (orange juice 80 ml, cream 30 g, sugar 20 g, vegetable oil 5 g).
- ***Supper***. Green salad, low-protein bread, butter, tea..
  - (potato 100 g, vegetable oil 15 g, lard 10 g, onion 20 g, ½ egg, green salad 30 g,; low-protein bread 40 g, butter 10 g, sugar 15 g.)
- ***Energy****2457 kcal/10,283 kJ; protein – 25 g, fat – 124 g, carbohydrates – 297 g, sodium – 1.49 g, potassium – 2.3 g, calcium – 0.25 g, phosphorus – 0.5 g.*

Day 3

- ***Breakfast 1.*** Bread, butter, honey, marmalade, coffee.
  - (low-protein bread 80 g, butter 20, marmalade 20 g, honey 10 g, cream 10 ml, sugar 10 g).
- ***Breakfast 2.*** Bread, lard with onion, red beet, tea.
  - (low-protein bread 40 g, lard 15 g, onion 5 g, red beet 30 g, sugar 10 g).
- ***Dinner.*** Potato, omelet, cauliflower, raspberry compote.
  - (potato 250 g, 1 small egg, vegetable oil 10 ml, cauliflower 100 g, butter 15 g, potato stretch 10 g, raspberry compote 150 ml).
- ***Lunch.*** Toast, butter, cream, coffee or tea.
  - (low-protein toast 40 g, butter 15 g, cream 10 g, sugar 5 g).
- ***Supper***. Bread, butter, sausage, orange, tea.
  - (low-protein bread 40 g, butter 20 g, sausage 10 g, orange 120 g, sugar 10 g).
- ***Energy****2458 kcal/10,285 kJ; protein – 25 g, fat – 130 g, carbohydrates – 285 g, sodium – 1.545 g, potassium – 2.26 g, calcium – 0.24 g, phosphorus – 0.43 g.*
